# Onychomycosis: A Review

**DOI:** 10.3390/jof1010030

**Published:** 2015-03-27

**Authors:** Bianca Maria Piraccini, Aurora Alessandrini

**Affiliations:** Division of Dermatology, Department of Experimental, Diagnostic and Specialty Medicine, University of Bologna, Via Massarenti 1, 40138 Bologna, Italy; E-Mail: aurora.alessandrini@alice.it

**Keywords:** onychomycosis, nail lacquers, systemic antifungal therapy, fungi, nail

## Abstract

Onychomycosis is the most common nail infective disorder. It is caused mainly by anthropophilic dermatophytes, in particular by *Trichophyton rubrum* and *T. mentagrophytes* var. *interdigitale*. Yeasts, like *Candida albicans* and *C. parapsilosis*, and molds, like *Aspergillus* spp*.*, represent the second cause of onychomycosis. The clinical suspect of onychomycosis should be confirmed my mycology. Onychoscopy is a new method that can help the physician, as in onychomycosis, it shows a typical fringed proximal margin. Treatment is chosen depending on the modality of nail invasion, fungus species and the number of affected nails. Oral treatments are often limited by drug interactions, while topical antifungal lacquers have less efficacy. A combination of both oral and systemic treatment is often the best choice.

## 1. Introduction

Onychomycosis is the most common nail infective disorder, and it is responsible for about 50% of all consultations for nail disorders. Onychomycosis has been reported as a gender- and age-related disease, being more prevalent in males and increasing with age in both genders [1]. In the elderly, onychomycosis may have an incidence >40% [2]. Predisposing factors are diabetes mellitus, peripheral arterial disease, immunosuppression due to HIV or immunosuppressive agents [3].

In most cases, this infection is caused by anthropophilic dermatophytes, in particular by *Trichophyton rubrum*, followed by *Trichophyton mentagrophytes* var. *interdigitale*. The non-dermatophyte molds, like *Scopulariopsis brevicaulis* and *Aspergillus* spp*.*, can be involved in onychomycosis as primary pathogens or as contaminant agents and secondary pathogens [4]. Other molds that have been isolated from affected nails include *Fusarium* spp*.*, *Acremonium* spp*.*, *Alternaria* spp*.* and *Neoscytalidium* sp. The estimated worldwide prevalence of non-dermatophyte molds onychomycosis is 10%–15% [5]. Yeasts, like *Candida* *albicans* and *Candida parapsilosis*, represent the third cause of nail fungal infection, and they occur only when predisposing factors are present, mainly immunosuppression and diabetes [6].

Toenails are more commonly affected than fingernails: onychomycosis in these cases frequently involves several nails, and dry-type plantar tinea pedis is often present [7]. There are different clinical types of onychomycosis, depending on the modality of nail invasion. Clinical diagnosis of onychomycosis always requires laboratory confirmation, and treatment depends on many factors, like the fungus species and the number of affected nails.

Onychomycosis in childhood is rare and affects approximately 0.5% to 2.6% of all children [8]. Similar to adults, the most common presentation is distal subungual onychomycosis, and toenails are affected more commonly than fingernails. Children acquire the fungus from a dystrophic or traumatic nail abnormality or from a parent, indirectly, through environment contamination [9]. Genetic predisposition to develop fungal invasion of the soles and nails seems necessary at a young age [10].

## 2. Clinical Features

### 2.1. Distal and Lateral Subungual Onychomycosis

**Figure 1:**
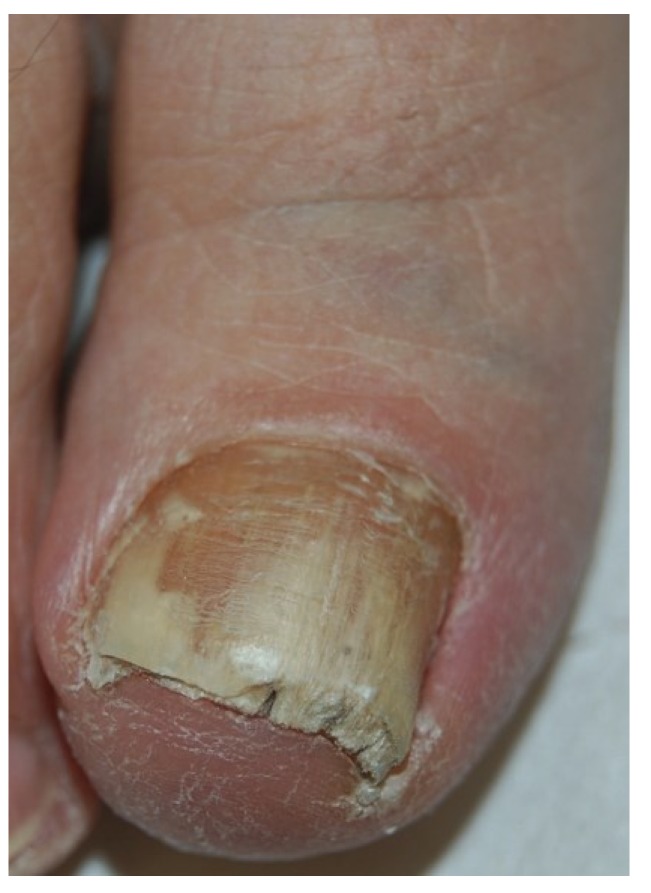
Distal and lateral subungual onychomycosis (DLSO): whitish discoloration, onycholysis and subungual hyperkeratosis.

**Figure 2:**
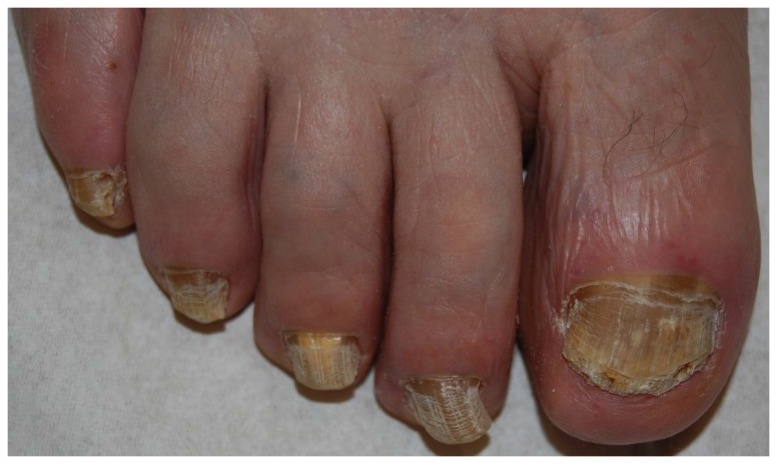
DLSO with prevalent yellow discoloration.

**Figure 3:**
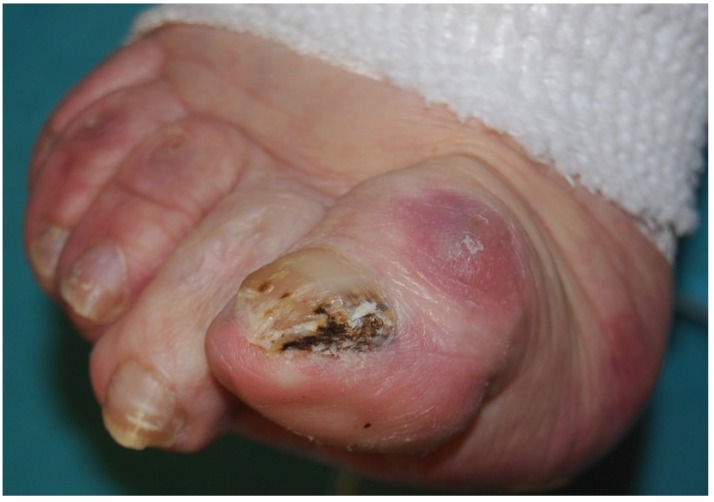
Pigmented DLSO.

**Figure 4:**
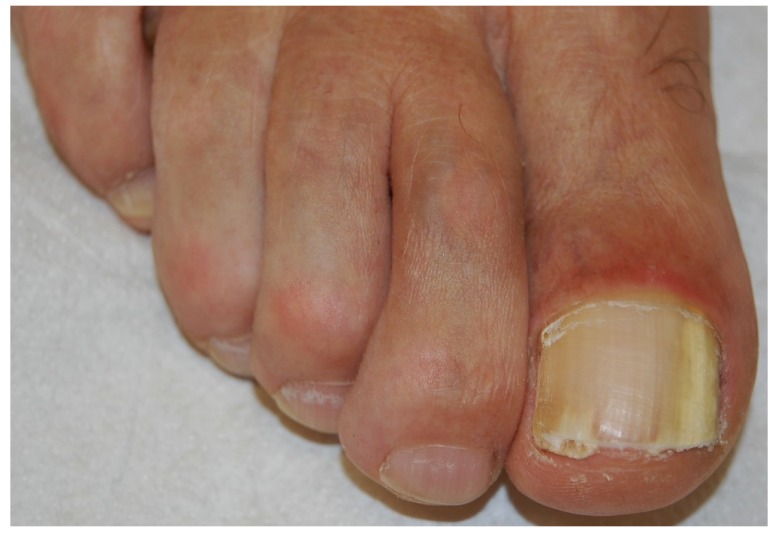
Onychomycosis due to molds, presenting the typical periungual inflammation.

Fungi reach the nail through the hyponychium and invade the undersurface of the nail unit plate spreading proximally. Distal and lateral subungual onychomycosis (DLSO) usually affects one or both of the great toenails and is also usually associated with tinea pedis [7]. The nail plate appears yellow-white, is detached due to onycholysis, with distal subungual hyperkeratosis (Figure 1). Less frequently, a brown, black or orange discoloration of the onycholytic nail can be seen (Figure 2). A possible presentation of DLSO due to dermatophytes is dermatophytoma, a subungual accumulation of hyphae and scales, scarcely reached by antifungals, which require excision of the area and systemic treatment. DLSO may be associated with black pigmentation of the nail (“fungal melanonychia”) (Figure 3), when the pathogen is the Melanoides variant of *Trichophyton rubrum* or other fungi that produce melanin, like *Neoscytalidium dimidiatum* or *Aspergillus niger* [11]. Onychomycosis due to non-dermatophytes is typically associated with a marked periungual inflammation (Figure 4). Differential diagnoses of DLSO include traumatic onycholysis (usually symmetrical and subungual hyperkeratosis is absent) and nail psoriasis (diffuse hyperkeratosis, several/all toenail involved, others skin and nail signs of psoriasis).

### 2.2. White Superficial Onychomycosis

**Figure 5:**
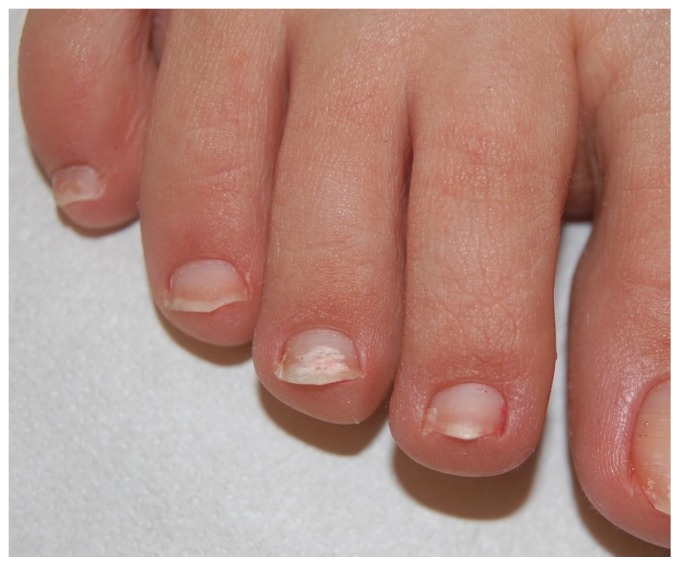
White superficial onychomycosis (WSO): white opaque friable patches of the nail plate.

Fungi invade the dorsal nail plate and form colonies that appear as white opaque formations, easily scraped away. The classical form is due to *Trichophyton interdigitale*, where dermatophytes colonize the most superficial layers of the nail plate without penetrating it (Figure 5), but *Fusarium* spp*.* and other molds may cause a white superficial onychomycosis (WSO) with a deeper nail invasion [12,13].

**Figure 6:**
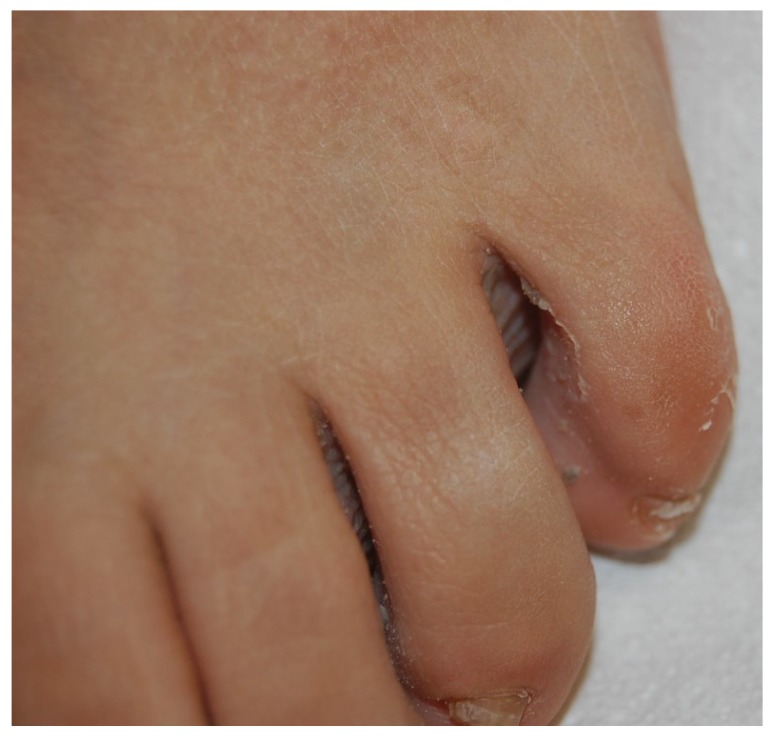
Tinea pedis interdigitalis, often associated with WSO.

Tinea pedis interdigitalis (athlete’s foot) due to *T. interdigitale* is common [7] (Figure 6).

Differential diagnosis includes superficial nail fragility due to prolonged wearing of nail polish and transverse toenail leukonychia due to trauma.

### 2.3. Proximal Subungual Onychomycosis

**Figure 7:**
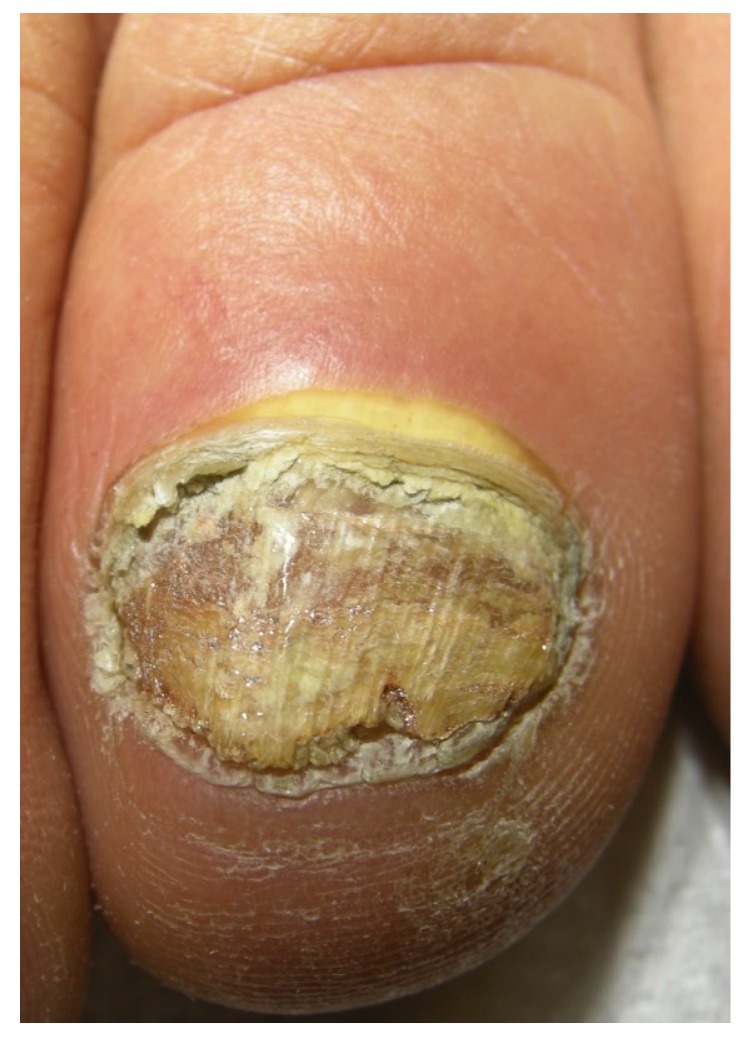
Proximal subungual onychomycosis (PSO): white discoloration of the proximal nail plate.

Fungal elements are typically located in the ventral nail plate, producing a proximal leukonychia. Proximal subungual onychomycosis (PSO) due to dermatophytes is very rare, and in the past, the form due to *T. rubrum* was considered as a sign of HIV infection. It presents as a white area under the proximal nail plate, in the lunula area (Figure 7). PSO is a common presentation of non-dermatophyte mold infection, especially due to *Aspergillus* sp. and *Fusarium* sp., and acute periungual inflammation is often associated. Differential diagnosis includes acute bacterial paronychia and pustular psoriasis of the nail.

### 2.4. Endonyx Onychomycosis

**Figure 8:**
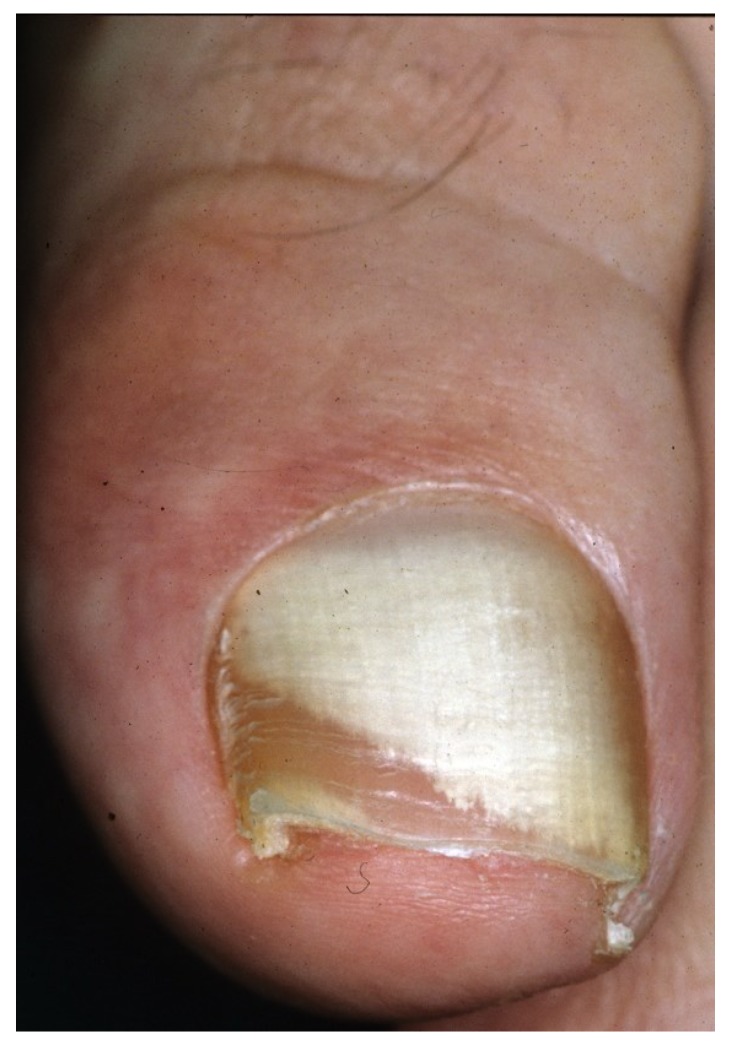
Endonyx onychomycosis: white discoloration of the nail plate that is firmly attached to the nail bed.

Endonyx onychomycosis is characterized by massive nail plate invasion in the absence of nail bed involvement. Clinically, the affected nail may show lamellar splitting and a milky white discoloration. The nail plate is firmly attached to the nail bed, and there is no nail bed hyperkeratosis or onycholysis [14] (Figure 8). This type of infection is very rare and caused by *T. soudanense* or *T. violaceum.*

### 2.5. Total Dystrophic Onychomycosis

**Figure 9:**
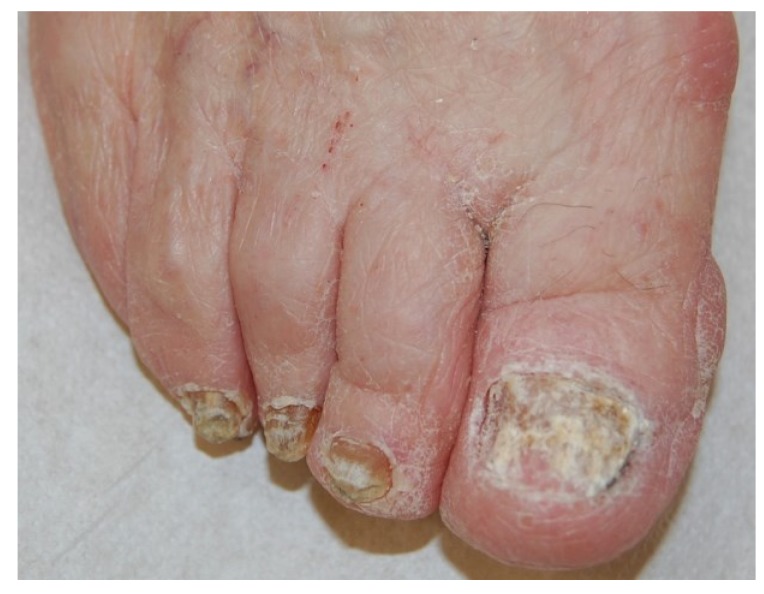
Total onychomycosis: the nail plate is completely invaded by fungi and friable.

Total dystrophic onychomycosis (TDO) is the most severe stage of onychomycosis, and it can result from a long-standing DLSO or PSO. The nail plate is diffusely thickened, friable and yellowish (Figure 9).

## 3. Diagnosis of Onychomycosis

The clinical suspect of onychomycosis should be confirmed by mycology. The mycological examination is composed by two parts: direct microscopic exam and culture. For the first one, the nail material, previously collected from the affected nail and immersed in a solution of KOH 40%, is put on a slide and then observed under the optical microscope to look for hyphae and spores. KOH does not allow one to recognize the type of fungus causing the onychomycosis, and a culture is needed for a more specific diagnosis. The histopathology of nail clippings can be utilized for diagnosing onychomycosis, with periodic acid-Schiff (PAS) stain that allows easy visualization of fungal hyphae.

Digital dermoscopy, also called onychoscopy, is an easy and quick procedure that allows differential diagnosis of onychomycosis from the common nail dystrophies.

**Figure 10:**
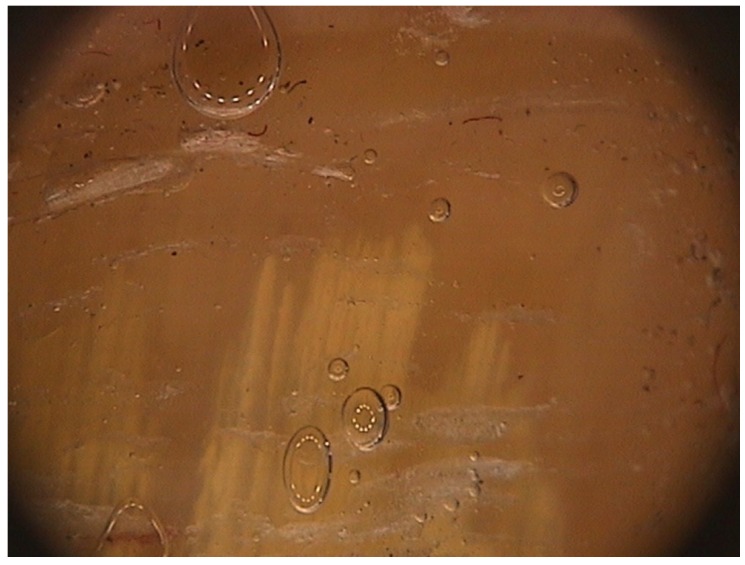
Onychoscopy of DLSO, showing the typical proximal fringed (ragged) margin.

The peculiar features of DLSO, not seen on traumatic onycholysis and nail psoriasis, are [15]: (1) proximal margin of the onycholytic area showing jagged edge, with sharp structures, directed to the proximal fold (Figure 10); (2) longitudinal striae of different colors in the onycholytic nail plate; and (3) the overall appearance of the color of the affected nail plate in a matted variable discoloration resembling the aurora borealis.

Confocal laser-scanning microscopy (CLSM) is an emerging diagnostic technique [16]. The aspect of dermatophytes appears as a network of lengthy structures with high reflection and the typical shape of hyphae: the CLSM aspect of yeasts has been reported by Arrese *et al*. [17], while molds have not been described yet in nails.

Some other interesting new tools in the diagnosis of onychomycosis are: dermatophyte test strip, fluorescence microscopy and Raman spectroscopy. The dermatophyte test strip is an immunochromatography test that uses a monoclonal antibody that reacts with *Trichophyton* species and gives a positive signal when in contact with one of these dermatophytes, after 15 min. It is a ready-to-use kit, very quick, easy to perform and not expensive. The test has a high sensitivity and negative predictive value, so it can be used to rule out onychomycosis in all doubtful cases. The technique had already been tried in a small series on onychomycosis [18].

Fluorescence microscopy consists of examining under fluorescent microscope nail clippings from suspected onychomycosis stained with PAS [19]. This method does not allow one to distinguish between the different species of fungi or between alive or dead hyphae, but it has lower cost than PAS stain. The disadvantages are the need for training and experience, the difficulties in the interpretation of false positives from true fungal fluorescence and the lower specificity compared with PAS and other special stains.

Raman spectroscopy is a vibrational spectroscopic technique that allows the investigation of the molecular composition of samples based on the molecular specificity of spectral bands in a vibration spectrum. Smijis and colleagues [20] showed only preliminary results, as the study was performed on nail clippings infected by fungi *in vitro*.

## 4. Treatment

Treatment of onychomycosis depends on the clinical type, the number of involved nails and the severity of the infection. The disadvantages of therapies are that oral treatments are often limited by drug interactions and potential hepatotoxicity, while topical antifungals have a limited efficacy if used without nail plate debridement. A combination of both oral and systemic treatment is often the best choice.

### 4.1. Topical Treatment

Penetration of a topical antifungal through the nail plate requires a vehicle that is specifically formulated for transungual delivery. The poor nail unit penetration limits the use of topical antifungal agents, and relapses and re-infections are common, occurring in at least 20%–25% of patients [21]. A combination with systemic antifungals, debridement or nail avulsion in severe onychomycosis reduces the duration of treatment and increases the cure rate [22]. Nail lacquers are effective in monotherapy in the treatment of WSO and of DLSO limited to less than 50% of the distal nail [23]. Treatment duration is 6–12 months. Possible options include amorolfine 5% or ciclopirox 8% in non-water-soluble lacquers and ciclopirox in water-soluble nail lacquer. Amorolfine nail lacquer is applied once a week, while ciclopirox nail lacquer is applied daily [24]. Amorolfine has fungistatic and fungicidal properties against dermatophytes, non-dermatophytes molds and yeast [25]. According to Gupta *et al*. [26], amorolfine 5% nail lacquer is recommended for onychomycosis without matrix involvement and mild cases of distal and lateral subungual onychomycosis that affect up to two nails.

Ciclopirox has fungicidal, anti-inflammatory and anti-allergic activity. It is applied daily. Two formulations exist: ciclopirox 8% in non-water-soluble lacquers and ciclopirox in water-soluble nail lacquer, which improved the nail permeability [26].

Efinaconazole 10% solution and tavaborole 5% solution are new topical antifungals for the treatment of dermatophyte-induced onychomycosis. Efinaconazole 10% nail solution is a promising drug, approved by the FDA in June 2014, for toenail onychomycosis [27]. It is a new triazole antifungal developed for topical treatment of mild to moderate DLSO, applied once daily without nail debridement. Cure rates are comparable to those seen with oral itraconazole [28]. A recent study [29] evaluated the efficacy of this nail lacquer on 1655 patients with onychomycosis for a period of 52 weeks, finding that efinaconazole was more effective at treating the early stage of the disease.

Tavaborole is formulated as a lightweight, water-soluble topical nail lacquer for the treatment of toenail onychomycosis [30]: it has received its first global approval for this indication in the U.S. [31]. Tavaborole 5% solution demonstrated efficacy and safety in phase 2 of clinical studies [32], but results from completed phase 3 studies are needed to provide additional evidences.

Terbinafine nail solution and a terbinafine spray, labeled TDT 067, may be good treatment alternatives in the future [33,34]. Other formulations with terbinafine that are undergoing phase 2 trials include MOB-015 and TMI-358 [35].

Luliconazole is an imidazole molecule with fungicidal and fungistatic activity, which has completed phases 1 and 2a for the treatment of moderate to severe distal subungual onychomycosis with positive results [36]. A phase 2b/3 of the study is still ongoing, with topical application of luliconazole at 10% with excellent tolerability and a safe profile [37].

Photosensitizers for photodynamic therapy (PDT) and a new laser system are emerging therapeutic options [38,39]. PDT involves the use of a photosensitizer and a light source that together generate reactive oxygen species, leading to chemical destruction of nail fungi [40]. It has been shown effective against many species of fungi, like *T. rubrum*. The most commonly-used photosensitizers are: phenothiazine dyes (methylene blue and toluidine blue), porphyrins, 5-Aminolevulinic acid (ALA) and methyl-aminolevulinate (MAL). A recent review [41] collected a total of six articles regarding the use of PDT in onychomycosis *in vivo,* but there are only case reports with small numbers of patients, except for two clinical trials. This study suggests that a previous nail abrasion or maceration (for example, with 20% urea ointment in occlusion) is needed prior to photosensitizer application. A limit of PDT is the high number of sessions: generally three to 12 are requested. The numbers of sessions could be reduced by increasing the amount of irradiation, but with more adverse side effects, like transient pain and burning. The optimal light source and number/frequency of treatments are not established yet, so further clinical trials are needed to assess a standardized method.

Other alternative treatments for onychomycosis include lasers, like the carbon dioxide laser, the Nd:YAG laser and the diode 870-nm, 930-nm laser (all approved by the FDA, for improvement of the cosmetic appearance of the nail and not for mycological cure), due to their minimally-invasive nature and the few number of requested treatment sessions. The carbon dioxide laser is the oldest laser and is infrequently used today thanks to the advent of less invasive lasers. With the Nd:YAG laser, small clinical trials have demonstrated mycological cure rates as high as 87.5% [42]. A recent study [43] demonstrated that the efficacy of long-pulsed Nd:YAG 1064 nm against affected toenails is superior to that against fingernails. The diode laser has shown mycological cure rates as high as 38% reported at the nine-month follow up [44], with minimal to no side effects [45]. A complete systemic review [46] investigated the use of lasers on onychomycosis, including a total of 12 published papers: two randomized controlled trials; four comparative design studies (with no placebo/control group); and the others were case series, investigating in the majority of cases (10/12) the 1064-nm neodymium laser.

The author concluded that there is no consensus on laser effectiveness, due to the heterogeneity of the study designs (the definition of “cure”, duration of the study, type of onychomycosis). To date, there were no studies comparing laser with traditional therapies for onychomycosis; more information is required for a better understanding of the efficacy of this treatment.

### 4.2. Systemic Treatment

DLSO extending to the proximal nail, PSO due to dermatophytes and deeply infiltrating white superficial onychomycosis require a systemic treatment. Fluconazole, itraconazole and terbinafine have improved treatment success [47], producing a mycological cure in more than 90% of fingernail infections and in about 80% of toenail infections. The reasons for treatment failures include the clinical characteristics of the onychomycosis (total onychomycosis, very thick subungual hyperkeratosis and dermatophytoma, which make it difficult for the drug to reach the affected are in active concentration), etiological agents (several non-dermatophytes do not respond to systemic antifungals, including *Neoscytalidium*, *Scopulariopsis* and *Fusarium* sp.), and patients comorbidities (immunodepressed patients have a poor prognosis, and several drugs may modify antifungal blood levels).

Terbinafine can be administered as a continuous therapy at 250 mg per day for 12 weeks or as a pulse therapy at the dosage of 500 mg/day for four weeks on and four weeks off [48]. Itraconazole is administered in pulse therapy at the dosage of 400 mg daily for one week a month. Treatment duration is two months for fingernails and three months for toenails.

Both continuous terbinafine and itraconazole pulse therapy are effective and safe in the management of dermatophyte toenail onychomycosis in people with diabetes [49]. These regimens can be associated with topical nail lacquers. There are no studies that evaluate the cure rates of combined therapies with systemic and topical antifungals, but these associations are commonly prescribed in clinical practice. Periodic removal of the affected nail plate, done by a podiatrist or by topical application of urea ointment, can accelerate improvement. Recurrences and reinfection are not uncommon (up to 20% of cured patient).

Fluconazole is also used in dermatophyte onychomycosis at the dosage of 150–300 mg weekly for more than six months, but is less effective [50]. Fluconazole, itraconazole and terbinafine have a good safety profile [47]. Posaconazole and albaconazole are new drugs that could be alternative therapy options [51,52].

In general, non-dermatophyte molds do not respond to systemic antifungals, and in these types of onychomycosis, the best choice is topical therapy associated with periodic removal of the affected nail plate. If the onychomycosis results caused by *Candida* sp., the drug of choice should not be terbinafine, as the yeast is not sensitive to it. Moreover, the isolation of *Candida* from a nail should always suggest a careful evaluation of the patient, as *Candida* onychomycosis is frequently associated with diabetes or immunodepression.

In cases of lateral nail plate involvement, dermatophytomas or dystrophic onychomycosis, surgical or chemical avulsion of the nail plate combined with topical or systemic treatment with itraconazole and terbinafine can be necessary [53].

Treatment of onychomycosis requires several month, as the nail grows very slowly, especially in the elderly. Drug choice relies on the type and severity of onychomycosis and the patient’s comorbidities. In the majority of the cases, patients present with a DLSO due to dermatophytes involving the distal part of one or two great toenails, and the treatment of choice is topical application of antifungals, possibly associated with periodic removal of the affected nail plate.

## 5. Conclusions

Onychomycosis is a very common fungal infection, which needs a targeted treatment. Therapy requires several month, as the nail grows very slowly, especially in the elderly. Drug choice relies on the type and severity of onychomycosis and the patient’s comorbidities. In the majority of the cases, patients present with a DLSO due to dermatophytes involving the distal part of one or two great toenails, and the treatment of choice is topical application of antifungals, possibly associated with periodic removal of the affected nail plate. For DLSO extending to the proximal nail, PSO due to dermatophytes and deeply infiltrating white superficial onychomycosis we recommend systemic treatment with fluconazole, itraconazole or terbinafine. Further studies on lasers and photodynamic therapy are needed before use can be standardized.
